# Congenital Dropsy—(Ascites)

**Published:** 1846-12

**Authors:** 


5. *Congenital Dropsy—(Ascites.)*—This case was that of a female child nine weeks old. I saw it October 25th, 1844, when the mother informed me that, at its birth, the abdomen seemed to be unusually protuberant, and that shortly afterwards it became subject to paroxysms of restlessness and crying, for which anodynes were administered without producing any relief. At my examination the general system was not emaciated, nor was there any anasarca of the extremities. The skin was rather soft and moist. It sucked heartily, had more thirst than natural, and had a slight coat upon the tongue. The abdominal tumor was so great as to extend down over the pubis, and also upwards and backwards over the ensiform cartilage and false ribs.

I prescribed diuretic and purgative medicines, and directed iodine ointment to be rubbed on the abdomen twice a day. No amendment followed this prescription. The child fell into the hands of another physician, who tapped it and drew off a considerable quantity of water. It eventually, however, died. —*Ibid.*

